# Regorafenib for recurrent high-grade glioma: a unicentric retrospective analysis of feasibility, efficacy, and toxicity

**DOI:** 10.1007/s10143-022-01826-z

**Published:** 2022-06-20

**Authors:** Hannes Treiber, Christian von der Brelie, Vesna Malinova, Dorothee Mielke, Veit Rohde, Claudia Ilse Chapuy

**Affiliations:** 1 Department of Hematology and Medical Oncology, University Medicine Göttingen, Robert-Koch-Straße 40, 37075 Göttingen, Germany; 2 Department of Neurosurgery, University Medicine Göttingen, Göttingen, Germany

**Keywords:** Glioblastoma, High-grade glioma, Regorafenib, Recurrence, Adverse events, CTCAE

## Abstract

We describe here 11 consecutive patients with recurrence of high-grade glioma treated with regorafenib at our university medical center. The majority of patients had MGMT promoter methylation (9/11 cases). Regorafenib was given as 2nd line systemic treatment in 6/11 patients and 3rd or higher line treatment in 5/11 patients. The median number of applied cycles was 2 with dosage reductions in 5/11. Response to treatment was observed in 4/11 (PR in 1/11, and SD in 3/11). Median overall survival for the cohort was 16.1 months, median progression-free survival 9.0 months, and median time to treatment failure 3.3 months. Side effects of any CTCAE grade were noted in all patients, hereby 6/11 with CTCAE °III-IV reactions. High-grade side effects were of dermatologic, cardiovascular, and hematologic nature. A mean treatment delay of 57.5 days (range 23–119) was noted between tumor board recommendation and treatment initiation due to the application process for off-label use in this indication. In conclusion, treatment with regorafenib in relapsed high-grade glioma is a feasible treatment option but has to be considered carefully due to the significant side effect profile.

**Supplementary Information:**

The online version contains supplementary material available at 10.1007/s10143-022-01826-z.

## Introduction

Brain tumors are a clinically and pathogenetically heterogeneous group of neoplasms among which glioblastoma stands out with an especially poor prognosis (2-year survival 26.5%) [13]. Despite the use of multimodal therapeutic approaches, outlook for glioblastoma patients has not changed significantly over the last 10 years. Standard 1st line therapy of glioblastomas is based on 4 pillars: surgical resection (if feasible gross total resection) followed by radiotherapy with concomitant chemotherapy (usually with temozolomide) and additional use of alternating electric fields (TTFields). Most studies aiming to improve prognosis over the past 15 years were disappointing. Only intensification of chemotherapy in a molecular subset of glioblastomas exhibiting methylation of the O-6-methylguanine-DNA methyltransferase (MGMT) promoter has shown moderate improvement in prognosis [7]. Despite optimal therapy, the disease invariably recurs; for example, in the seminal work that defined the current standard of care, the median progression-free survival was 6 months [13].

The treatment of patients with recurrence or progression of high-grade glioma, e.g., °III or °IV tumors according to the WHO 2016 classification, presents an interdisciplinary challenge because of limited treatment options and due to the lack of therapeutic standards. Depending on the location and extent of the tumor, second surgery can be pursued, but may not be an option for all patients [18]. The feasibility and efficacy of repeat radiotherapy are critically discussed [18]. Systemic treatment with chemotherapy or targeted therapy is another challenge. Methylation of the MGMT promoter suggests efficacy of retreatment with temozolomide, although the median time to treatment failure in this situation is 3.2 months [17]. Lomustine (CCNU) has emerged as an alternative option, although responses were of short duration in many studies with progression-free survival ranging from 1 month to 82 days [1, 14, 16]. Additional therapy with bevacizumab improved progression-free survival but did not prolong overall survival in patients with recurrent glioblastoma [19] and was therefore not approved for this indication in the European Union.

Regorafenib represents a new oral treatment option for these patients in high demand of another effective line of treatment. Regorafenib is a multi-tyrosine kinase inhibitor targeting angiogenesis, cell proliferation, and tumor stroma including VEGFR1-3, TIE2, KIT, RET, RAF1, BRAF, PDGFR, and FGFR. To date, regorafenib is used for the treatment of advanced metastatic colorectal carcinoma [6], as a 2nd-line therapy for hepatocellular carcinoma [2] and as 3rd-line treatment for gastrointestinal stromal tumor [4]. In the multicenter phase II REGOMA trial in patients with recurrence or progression of glioblastoma after 1st-line therapy with surgery and radiochemotherapy, regorafenib was compared with the current standard of care, lomustine [10]. This showed a significant advantage in median overall survival in favor of regorafenib of 7.4 months versus 5.6 months with lomustine therapy [10]. Surprisingly, the median OS for the lomustine arm was rather low compared to other trials, where 8 to 9.8 months median OS was reported for treatment with lomustine [1, 14, 19]. Further data from retrospective studies support efficacy of regorafenib in glioblastoma [11, 15]. The biggest retrospective analysis on 54 patients treated with regorafenib in glioblastoma reports an even longer median OS of 10.2 months [11].

Known therapy-limiting side effects with regorafenib include the occurrence of hand-foot skin reaction (HFSR), arterial hypertension, and elevations in lipase and bilirubin, whereas with lomustine therapy, hematologic toxicity is a particularly prominent side effect [10]. The same side effect profile was reported with regorafenib treatment in glioblastoma, but occurrence of °III and IV HFSR, hypertension, and fatigue was lower as described for patients with colorectal carcinoma in CORRECT trial [6, 10, 11]. It is expected, that these side effects occur more frequently, when regorafenib is used after several lines of systemic treatment compared to patients in the REGOMA trial who were treated in 2nd line with regorafenib.

Treatment with regorafenib represents another option for patients with good performance status who have already undergone standard lines of therapy. Since treatment with regorafenib is currently not approved by the European Medicines Agency for high-grade glioma, it can only be used after application and approval by the health insurance company. No data on the logistics of regorafenib treatment in glioma patients are available, which is important in states like Germany where regorafenib can only be prescribed after approval of the health insurance company of the patient. Therefore, the aim of this study was to obtain real-world data on feasibility, efficacy, and toxicity of regorafenib in high-grade glioma patients.

## Methods

This retrospective, single-center observational study describes the course of patients with recurrent high-grade glioma treated with regorafenib at the University Medical Center of Göttingen, Germany. The study was approved by the local institutional review board.

Included were all patients with diagnosis of histologically proven recurrent high-grade glioma (°III–IV according to WHO 2016) independent of IDH and MGMT promoter status and evidence of progressive disease on imaging according to RANO criteria, who received regorafenib treatment since August 2019 at our institution. Data cut-off was March 31, 2021. All included patients were > 18 years of age and received after initial surgery first-line treatment according to the Stupp-protocol or CeTeG/NOA-09 protocol. Other chemotherapy as 2nd- or 3rd-line treatment before initiating regorafenib was allowed. Patients with previous anti-angiogenic treatment were not candidates for regorafenib and were thus excluded. Adequate bone marrow reserve (no presence of cytopenia CTCAE °III-IV) as well as appropriate liver and renal function were required before starting regorafenib. Patients with medical contraindications to regorafenib (e.g., uncontrolled hypertension, prior thrombo-embolic events) did not qualify for treatment with regorafenib.

Before initiating regorafenib treatment, all cases were discussed in our interdisciplinary tumor board. Because treatment with regorafenib in recurrent high-grade glioma is not approved by the EMA, the treating physician had then to apply for coverage of related health care cost with the patients’ health insurance. Treatment with regorafenib was only initiated after insurance approval. During treatment with regorafenib, patients were monitored at our interdisciplinary neuro-oncological clinic with neurosurgeons and oncologists.

Patients were treated with regorafenib at standard dose 160 mg once daily for the first 21 days of a 28-day cycle. Unacceptable toxicity leads to regorafenib dose reductions to 120 mg and 80 mg as described in REGOMA trial. During treatment, patients were instructed to have their blood work checked (basic metabolic profile and complete blood count) weekly for the first cycle and then biweekly. All patients were evaluated clinically in the office before initiation of the next treatment cycle. Adverse events were monitored and graded according to the National Cancer Institute Common Terminology Criteria for Adverse Events v5.0 (CTCAE) [21]. Treatment response was assessed with gadolinium brain MRI every 12 weeks.

### Analyzed data and statistics

Patient charts were analyzed retrospectively. For purpose of this study, we reviewed inpatient charts as well as outpatient clinic notes of the treating physician. The primary objective was to collect data about safety, toxicity, and treatment adherence. For this purpose, we collected demographic data, medical data of initial diagnosis and progress (date, histology, molecular profile of the tumor), and treatment data (surgery, radiochemotherapy with either temozolomide alone or temozolomide, and lomustine in combination plus optionally tumor-treating fields). Medical data concerning the diagnosis and treatment of recurrent disease was obtained. For toxicity analyses, we documented all clinical and laboratory adverse events graded by CTCAE. The secondary objective of this study was to obtain overall survival data defined as the time of treatment start with regorafenib until death due to any cause. The date of the tumor board decision to treat with regorafenib was used as starting point for survival analysis, further enabling analysis of treatment delay due to the health insurances approval process. For the primary outcome, survival data were analyzed by Kaplan–Meier methods. Log-rank test was used for univariate analysis, and *p*-values were considered significant with *p* ≤ 0.05. Statistical analyses were done using GraphPad Prism 9.

### Cohort characteristics

Overall, 11 patients with recurrent high-grade glioma received regorafenib treatment at our institution in the study period. The majority of patients were male (8 male, 3 female). Median age was 53 years. Pathological diagnosis was glioblastoma WHO °IV (2016) in 10 and astrocytoma WHO °III (2016) in 1 patient. IDH status was wild type in 8 (mutated in 3 patients), and MGMT promoter was methylated in 9 patients and non-methylated in 2 patients. The initial treatment was surgery followed by radiochemotherapy with temozolomide (Stupp) [13] in 5 patients and surgery followed by radiochemotherapy with temozolomide and lomustine (CeTeG/NOA-09 protocol) in 6 patients [7] (see Table 1). Performance status according to Eastern Cooperative Oncology Group (ECOG) was 2 or better in 10/11 patients.

**Table 1:** Patients characteristics (*ECOG* Eastern Cooperative Oncology Group Performance Status, *IDH* isocitrate dehydrogenase, *MGMT* O-6-methylguanine-DNA methyltransferase)

Characteristics	Patients (*n* = 11)
Gender	
Male	8
Female	3
Age	
Median (range)	53 (30–70)
ECOG	
0	2
1	5
2	3
3	1
Histology (WHO 2016)	
Glioblastoma	10
Astrocytoma WHO °III	1
IDH status	
Wild-type	8
Mutated	3
MGMT status	
Methylated	9
Unmethylated	2
Initial treatments	
Temozolomide (Stupp)	5
Temozolomide + lomustine (CeTeG/NOA09)	6
Median time from tumor board recommendation to regorafenib treatment (days)	57.5 (23–119)

## Results

### Regorafenib treatment

Regorafenib treatment was prescribed by the treating neuro-oncologist. The dosing regimen was 160 mg taken orally once a day for 3 weeks followed by a 1-week break before the next cycle. It was used as 2nd-line systemic therapy in 6 patients and as 3rd- or higher line of treatment in 5 patients. Five patients that received regorafenib as 2nd line had initial treatment with temozolomide and lomustine (CeTeG/NOA-09); 1 patient had temozolomide only (Stupp). Second surgery at time of relapse was performed in 6 of 11 patients prior to regorafenib initiation. Dexamethasone was started in 5 of 11 patients prior to regorafenib treatment. The median duration of treatment with regorafenib was 2 treatment cycles with a wide range between 1 and 12 cycles. Dose adjustments for regorafenib were employed in 5 of 11 patients (see Table 2).

**Table 2:** Regorafenib treatment (CTCAE = Common Terminology Criteria for Adverse Events)

Characteristics of regorafenib Treatment	Patients (*n* = 11)
Line of treatment	
2nd	6
3rd or higher	5
Duration of treatment Median Administered cycles	Days 2 (1–12)
Dose reduction	5
Best response to regorafenib	
Partial response	1
Stable disease	3
Progressive disease	4
Unknown	3
Reason discontinuation	
Progressive disease	3
Adverse event	6
Palliation	1
Unknown	1
Adverse Events (CTCAE)	
Any event	11
Grade 3–4 event	6
Hand-foot skin reaction	
Yes	5
No	5
Unknown	1
Corticosteroid use	
Yes	5
No	5
Unknown	1

Best responses to regorafenib treatment were partial remission in 1 patient, stable disease in 3 patients, and progressive disease in 4 patients. The patient achieving a partial remission (PR) as best response to regorafenib received the most cycles (12) of regorafenib treatment and had the longest overall survival (18 months) in our cohort. A detailed description of all patients can be found in the supplement (Table S1). No follow-up imaging was performed for 3 patients due to cessation of regorafenib treatment due to side effects in 2 cases and change of treatment goal to best supportive care in 1 case. Regorafenib treatment was discontinued due to progressive disease in 3 patients, serious adverse events in 6 patients, and change of treatment goal to best supportive care in 1 patient (see Table 2).

**Fig. 1:**
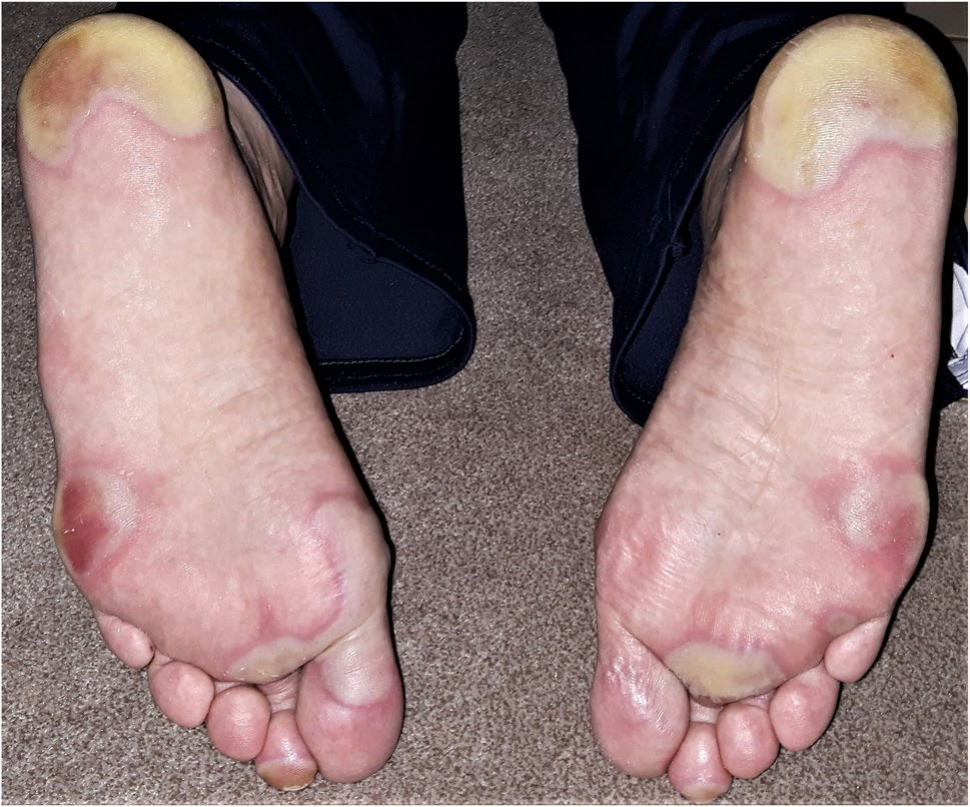
Severe hand-foot skin reaction (HFSR) in a patient with glioblastoma treated with regorafenib, leading to impaired activities of daily livings

All patients experienced treatment-related side effects. Severe adverse events (CTCAE °III-IV) occurred in 6/11 patients. In total, there were 8 adverse events °III or °IV. Severe adverse events were of dermatologic, vascular, and hematologic nature. HFSR occurred in 5/11 patients, with °III HFSR occurring in 2 patients (see Fig. 1). No °IV HFSR was noted. A thromboembolic event °IV in the form of a central pulmonary embolism was reported in 1 patient. Hypertension was noted in 4/11 patients, with 2°III and 2°IV events. Hematologic toxicity °III in the form of leucopenia occurred in 1 patient. Further adverse events were mild (°I–II) and were of dermatologic, hematologic, or serologic nature. Thrombocytopenia occurred in 7/11 patients, leucopenia °I–II in 2/11 patients, and anemia °I in 3/11 patients. Serologic abnormalities included alanine aminotransferase (ALT), bilirubin, gamma-glutamyl transferase (GGT), and lipase increases (°I–II, see Table 3). Fatigue was another treatment-related side effect observed in 6/11 patients.

**Table 3:** Adverse events during the study period by CTCAE (Common Terminology Criteria for Adverse Events) grade. (*ALT* alanine aminotransferase, *GGT* gamma-glutamyl transferase)

Event/CTCAE Grade	°1	°2	°3	°4
Hematological				
White blood cells decreased	1	1	1	0
Platelet count decreased	6	1	0	0
Anemia	3	0	0	0
Gastrointestal				
ALT inreased	3	0	0	0
Blood bilirubin increased	1	0	0	0
GGT increased	3	1	0	0
Lipase increased	1	0	0	0
Diarrhea	2	0	0	0
Skin				
Hand-foot skin reaction	1	2	2	0
Vascular				
Thrombosis	0	0	0	1
Hypertension	0	0	2	2

### Oncological follow-up and outcome

**Fig. 2:**
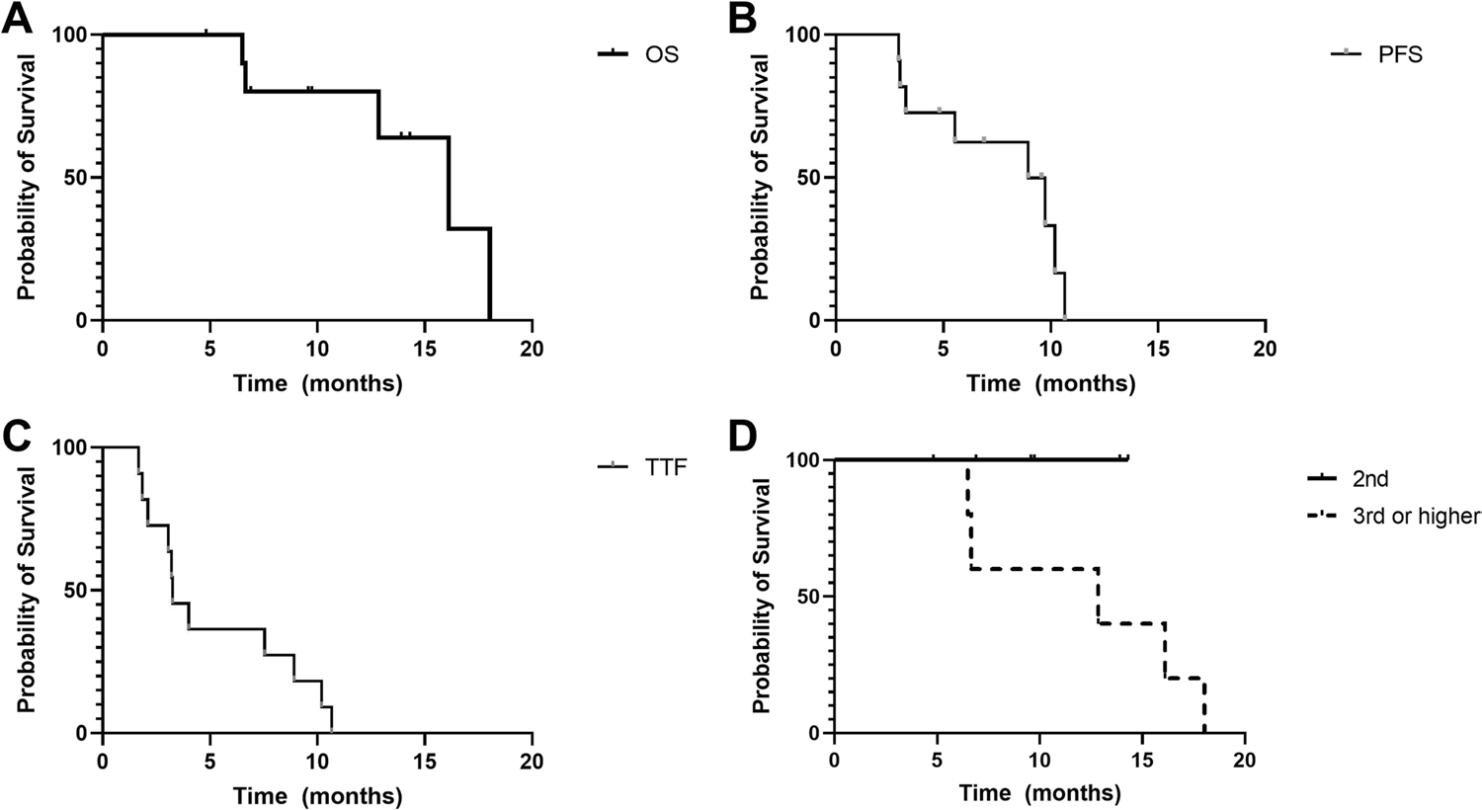
**A** Overall survival (OS) for the entire cohort. The median OS is 16.1 months. **B** Progression-free survival (PFS) for the entire cohort. The median PFS is 9.0 months. **C** Time to treatment failure (TTF) for the entire cohort. The medianTTF is 3.3 months **D** OS by application of regorafenib in 2nd versus 3rd or higher (Median OS undefined vs 12.9 months, *p* = 0.0900)

During the study period, 5 patients passed away. Overall survival (OS) for the entire cohort for treatment with regorafenib was 16.1 months, median progression-free survival (PFS) 9.0 months, and time to treatment failure (TTF) 3.3 months (Fig. 2A–C). Patients who received regorafenib in 2nd line versus 3rd or higher line of treatment showed a trend towards improved OS, without statistical significance (*p* = 0.0900) (Fig. 2D). Mean delay from tumor board recommendation to 1st application of the intended treatment was 57.5 days (range 23–119). There was no significant survival difference between the patients with early vs. late initiation of regorafenib treatment as dichotomized according to the median delay (*p* = 0.8236, Fig. S1A). Analysis of only IDH wild-type tumors (8 patients) demonstrates a median overall survival of 12.9 months in this subgroup (Fig S1B).

## Discussion

This retrospective analysis on 11 consecutive patients demonstrates three key findings. (I) A relatively favorable outcome for the whole cohort; (II) occurrence of side effects in all patients, with higher-grade events in 6/11 patients of dermatologic, cardiovascular, and hematologic nature; and (III) a significant delay of treatment initiation with a median of 57.5 days caused by the insurance approval process.

While the OS compares favorably to REGOMA trial [10], we think this may only be partially attributed to regorafenib treatment. A major confounding factor is likely the composition of the cohort. First, 3/11 patients in our cohort have IDH-mutated tumors, in contrast to the REGOMA trial with only 2/44 IDH mutated tumors in the regorafenib treatment arm [10]. After removal of the three IDH mutated patients from our cohort, median overall survival decreases from 16.1 to 12.9 months, reflecting the more aggressive biological behavior of these tumors. The longest overall survival after regorafenib therapy was achieved in a patient suffering from anaplastic astrocytoma (diagnosis confirmed by Illumina 450k methylation array). Moreover, a prominent proportion of our cohort consists of MGMT-methylated tumors, resulting in a high proportion of patients treated according to CeTeG/NOA-09 protocol in 1st line. In this regard, excellent disease control for regorafenib as 2nd-line treatment in a patient with MGMT methylated promoter was described in a case by Detti and colleagues [3]. However, follow-up in this case report was relatively short with only three cycles regorafenib administered [3]. In the REGOMA trial, MGMT promoter methylation status was evenly balanced in the regorafenib arm, with non-methylated MGMT promoter in 30/59 cases and methylated promoter in 29/59 cases [10]. A bicentric retrospective analysis on regorafenib for high-grade gliomas scrutinizing 24 patients reported a worse median OS than the REGOMA trial (7.4 months in the clinical trial vs. 4.1 months in the real-world situation). In this study, 17/24 tumors had a non-methylated MGMT promoter [15]. We hypothesize that the survival differences between these three studies may partly be explained by the difference in cohort composition regarding MGMT promoter methylation status, especially explaining the favorable outcome found in the cohort presented here. For interpretation of the outcome of the bicentric study, further factors are certainly central: this real-life study includes patients with lower Karnofsky performance status (50–60% in 6/24) and more advanced disease (only 3/24 patients treated with regorafenib at first relapse) as opposed to the REGOMA trial.

Our data demonstrate that the time to treatment failure, defined as death, progressive disease, or cessation of treatment due to other reasons (e.g. side-effects), is much lower than the progression-free survival, defined as progressive disease or death: median progression-free survival (PFS) is 9.0 months and time to treatment failure (TTF) 3.3 months. Our data suggest that this is driven mainly by the frequent cessation of therapy due to the side effects of regorafenib treatment, which was prominent in our cohort. In the case report cited above, side effects were not described [3]. The bicentric retrospective analysis provides no CTCAE grading for adverse events for this cohort, and only the occurrence of HFSR is reported in 8/24 patients, predicting significantly better overall survival [15]. This observation is in line with reports concerning tyrosine kinase inhibitors in other malignancies [9], but could not be repeated in our patient cohort, probably due to the small sample size. While the occurrence of pulmonary embolisms was reported in another retrospective cohort [20], this severe side effect did not occur in the original REGOMA trial, underlying the need for further real-world data. Another striking example in this regard is hypertension °III–IV, which occurred in a significant proportion of patients with hepatocellular or colorectal cancer patients treated with regorafenib [2, 6]. Similarly, in our case series, we found hypertension °III–IV in 4/11 patients, with 2 patients requiring intensive care monitoring. However, in the REGOMA trial, hypertension °III–IV occurred less frequently, only in 1 patient, and was not reported in the other glioma reports, while our cohort demonstrates that this is a significant side effect also in glioma patients with impact on quality of life and therefore should be monitored carefully and treated accordingly [10, 11, 15].

Due to the above-described side effects, we noted frequent cessation of regorafenib therapy (6/11 patients). In the REGOMA trial, 4/59 patients treated with regorafenib stopped the treatment due to side effects [10]. In the CORRECT trial, 500 patients with colorectal patients with colorectal cancer were treated with regorafenib, and treatment was stopped in 43 cases due to adverse events associated with disease progression and 42 cases due to adverse events not associated with disease progression [6]. In the RESORCE trial, 379 patients with hepatocellular carcinoma received regorafenib, and in 56 cases adverse events associated with disease progression,47 adverse events not associated with disease progression, 1 not further specified adverse event, and 26 withdrawals by patient’s decision were noted [2]. In a real-world setting, a small retrospective series analyzing 6 patients receiving regorafenib for high-grade astrocytoma found progressive disease as best radiographic response in all patients and adverse events °III in 5/6 patients [8].This demonstrates that even in clinical trials with highly selected, motivated patients, occurrence of side effects leading to treatment cessation is relatively high. This may further be aggravated in the more palliative situation of recurrent high-grade glioma, when patients and treating physicians aim to improve or maintain quality of live and survival in a well-balanced manner.

The delay between application for regorafenib therapy and actual treatment initiation is a major concern when selecting and counseling patients for this treatment option. Our data demonstrate that this interval is rather long, especially considering the life expectancy of patients with recurrent high-grade glioma. None of the other studies using regorafenib in glioma patients, provides data on the insurance approval process for the off-label use of regorafenib in this indication. [10, 11, 15]. The treating physician should take into consideration the long approval process of the drug when making the treatment decision.

This aspect touches the optimal patient selection for regorafenib treatment. We hypothesize that due to logistic concerns, especially patients with MGMT-methylated tumors may qualify for this option. On a biological level, a molecular analysis accompanying the REGOMA trial used genome-wide transcriptomics and microRNA profiles from tumor samples to determine a molecular signature associated with improved regorafenib response. This study found that the gene transcripts HI1A and CDKN1A and the microRNAs miR-3607-3p, miR-301a-3p, and miR-93-5p defined a subgroup with improved survival after regorafenib treatment [12]. While this approach is promising, it is not applicable to routine patient care at the moment.

### Limitations

Due to the retrospective nature of this study, there are major limitations. The oncologic follow-up was tailored to the patient’s specific situation, providing clinical care during outpatient visits phone consults or via e-mail communication. While this results in close patient-physician relation, it may decrease the amount of medical data available, ranging from blood work results to imaging studies in comparison to a structured trial. Therefore, the progression-free survival data is not optimal due the lack of stringent MRI scans in short intervals. While this is a shortcoming in comparison to a structured prospective clinical trial, it may well reflect routine oncology practice. Another limitation presents the small cohort of patients, which does not allow thorough statistical analysis. In this regard, we noticed a trend to improve overall survival for patients who received regorafenib as 2nd-line treatment which may have reached statistical significance in a bigger cohort. Another bias presents the high proportion of patients with MGMT methylated tumors, which are known to have a more favorable overall survival. The reasons are probably a preselection bias of patients with good performance status and the retrospective approach of this study. Furthermore, a recent study described a novel radiologic biomarker with prognostic relevance in glioma patients treated with regorafenib, distinguishing a classical progressive disease from a T2-dominant growth pattern [20]. Due to the low number of patients still under active regorafenib treatment when experiencing progression in imaging, we only have 4 imaging studies available in this regard, all showing a classical progressive disease.

## Conclusion

Observed response rates were lower compared to the randomized phase II trial (REGOMA) and are probably reflecting the different patient population with more advanced stage disease and higher number of relapses. The favorable overall survival may partly be attributed to the molecular composition of the cohort, with strong predominance of MGMT-methylated tumors. The potential long delay between application for regorafenib treatment and treatment initiation must be taken into consideration when selecting and counseling patients for this treatment.

In conclusion, treatment with regorafenib is a feasible treatment option for recurrent high-grade glioma patients; however, the significant side-effect profile and modest response rate must be considered carefully when choosing this option. The international phase II/III GBM AGILE trial will provide further insight in regorafenib treatment for glioblastoma [5].

## Supplementary Information

Below is the link to the electronic supplementary material.

Supplementary file1 (DOCX 70 KB)

## Data Availability

Not applicable.
