# Supplementary material for: Regorafenib for recurrent high-grade glioma: a unicentric retrospective analysis of feasibility, efficacy, and toxicity
Source: Neurosurg Rev. 2022 Jun 20;45(5):3201–8. doi: 10.1007/s10143-022-01826-z (PMC9492606; doi:10.1007/s10143-022-01826-z)
Supplement: Supplementary file 1 — Supplementary file1 (DOCX 70 KB) [file 10143_2022_1826_MOESM1_ESM.docx]

**Supplemental Table S1: Detailed anonymized description of individual patients.**

| **Patient** | **Initial Histology** | **IDH Status** | **MGMT Status** | **ECOG at regorafenib initiation** | **Best response to regorafenib** | **Administered cycles of regorafenib** | **Reason discontinuitation** | **OS in months** | **High-grade side effects (CTCAE °III-IV)** |
| --- | --- | --- | --- | --- | --- | --- | --- | --- | --- |
| **1** | GBM | WT | negative | 1 | na | 1 | side-effect | 6,89 | none |
| **2** | GBM | WT | methylated | 2 | PD | 5 | na | 6,49 | hypertension, HFSR |
| **3** | GBM | WT | methylated | 2 | SD | 1 | side-effect | 9,74 | hypertension |
| **4** | GBM | WT | methylated | 1 | na | 1 | side-effect | 4,82 | HFSR |
| **5** | GBM | WT | methylated | 3 | na | 1 | palliation | 9,57 | none |
| **6** | GBM | WT | methylated | 2 | PD | 2 | PD | 6,66 | hypertension |
| **7** | GBM | WT | methylated | 1 | PD | 4 | side-effect* | 12,85 | none |
| **8** | GBM | mut | methylated | 0 | SD | 6 | PD | 13,90 | none |
| **9** | Astro III | mut | methylated | 1 | PR | 12 | side-effect | 18,03 | hypertension, thrombosis |
| **10** | GBM | WT | negative | 0 | PD | 1 | side-effect | 16,10 | haematological |
| **11** | GBM | mut | methylated | 1 | SD | 8 | PD | 14,30 | none |
|  |  |  |  |  |  |  |  |  |  |
|  |  |  |  |  |  |  | *due to patients wish |  |  |

**Supplemental Figure S1A: Influence of treatment initiation delay on overall survival.**


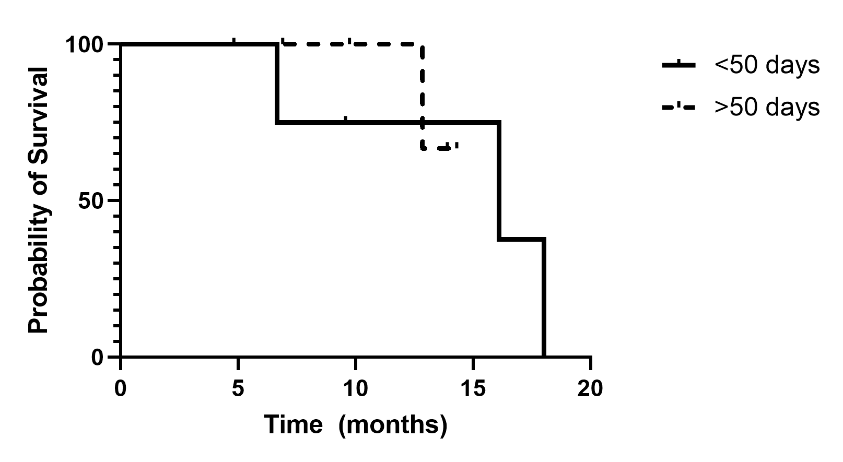


There is no statistically significant difference between early and late initiation in our cohort (p=0.8236).

**Supplemental Figure S1B: Survival of only IDH wildtype tumours (n = 8).**


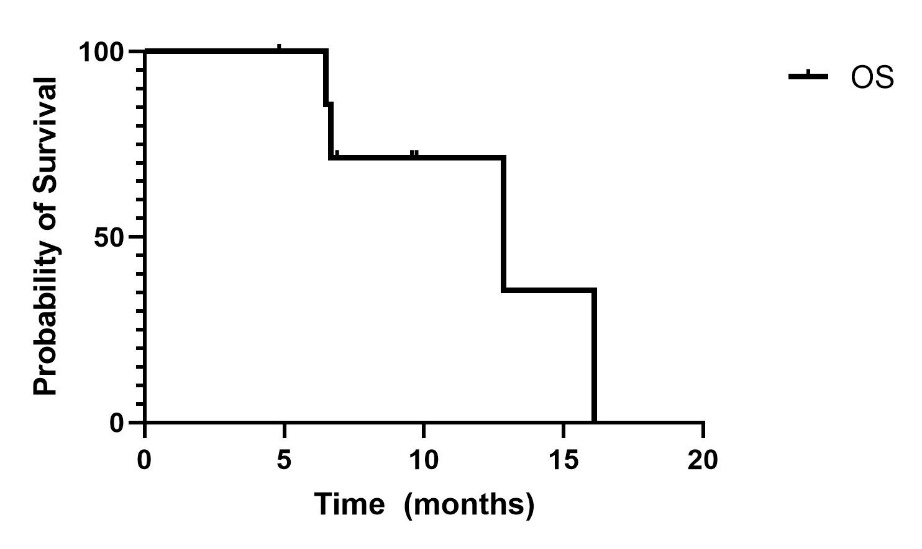


The median overall survival for this subgroup is 12.9 months.
